# Storytelling as a communication tool for health consumers: development of an intervention for parents of children with croup. Stories to communicate health information

**DOI:** 10.1186/1471-2431-10-64

**Published:** 2010-09-02

**Authors:** Lisa Hartling, Shannon Scott, Rena Pandya, David Johnson, Ted Bishop, Terry P Klassen

**Affiliations:** 1 Alberta Research Centre for Health Evidence, Department of Pediatrics, University of Alberta, Edmonton, Alberta, Canada; 2 Faculty of Nursing, University of Alberta, Edmonton, Alberta, Canada; 3 Stollery Children's Hospital, Edmonton, Alberta, Canada; 4 Departments of Pediatrics and Physiology & Pharmacology, University of Calgary, Alberta, Canada; 5 Department of English and Film Studies, University of Alberta, Edmonton, Alberta, Canada

## Abstract

**Background:**

Stories may be an effective tool to communicate with and influence patients because of their ability to engage the reader. The objective of this paper is to describe the development of a story-based intervention for delivery of health evidence to parents of children with croup for use in a randomized controlled trial.

**Methods:**

A creative writer interviewed parents of children with croup presenting to the pediatric emergency department (ED) and drafted stories. We revised the stories based on written participant feedback and edited the stories to incorporate research evidence and health information. An illustrator and graphic designer developed story booklets which were evaluated through focus groups.

**Results:**

Ten participants provided feedback on the five stories drafted by the creative writer. Participants liked the concept but found the writing overly sophisticated and wanted more character development and more medical/health information. Participants highlighted specific story content that they liked and disliked. The revised stories were evaluated through focus groups involving eight individuals. Feedback was generally positive; one participant questioned the associated costs. Participants liked the graphics and layout; felt that they could identify with the stories; and felt that it was easier to get information compared to a standard medical information sheet. Participants provided feedback on the story content, errors and inconsistencies, and preferences of writing style and booklet format. Feedback on how to package the stories was provided by attendees at a national meeting of pediatric emergency researchers.

**Conclusions:**

Several challenges arose during the development of the stories including: staying true to the story versus being evidence based; addressing the use of the internet by consumers as a source of health information; balancing the need to be comprehensive and widely applicable while being succinct; considerations such as story length, reading level, narrative mode, representation of different demographics and illness experiences, graphics and layout. The process was greatly informed by feedback from the end-user group. This allowed us to shape our products to ensure accuracy, credibility, and relevance. Our experience is valuable for further work in the area of stories and narratives, as well as more broadly for identifying and developing communication strategies for healthcare consumers.

## Background

There is a trend towards the use of narratives, stories, and storytelling in the healthcare setting as a tool for diagnostics [1,2], therapeutics [3-5], and the education of patients, students, and practitioners [6-11]. The effectiveness of storytelling as a communication tool has been supported by evidence from several disciplines including nursing, social science, and psychology [12]. An appeal of storytelling is its ability to present information couched within a personal account that engages the reader and validates their own experiences. There is evidence that memory of information may be enhanced when presented in narrative form.

The goal of our research program was to investigate storytelling as a tool to engage parents in communicating research and health information in order to affect parental and child outcomes, and healthcare utilization. We chose to examine storytelling in the context of the pediatric emergency department (ED) where parental anxiety may be high due to the medical condition of their child and the lack of certainty around what to expect in terms of diagnosis and management. Further, due to the intense pace of the ED setting, time spent with healthcare practitioners may be short. We chose croup as the condition with which to examine our hypothesis because of the frequency of its presentation to the ED, the anxiety that it causes for parents [13], and the large body of evidence that supports the therapeutic management of the disease [14-19]. Moreover, clinical practice guidelines for the diagnosis and management of croup are available (e.g., http://www.albertadoctors.org/bcm/ama/ama-website.nsf/AllDoc/87256DB000705C3F87256E05005534E2/$File/CROUP.PDF). These recommend the use of dexamethasone (all cases) and epinephrine (severe cases) according to defined protocols.

While there is a growing body of literature discussing the use of stories and storytelling as a communication tool in healthcare or health promotion, there are few accounts describing the development of the interventions including gathering feedback from the end-user group. While one study stressed the need for formative research, the authors commented that this is often not possible due to limited time and resources [20]. Others have observed that there is often a disproportionate amount of effort "into developing the narrative without understanding how it is received [21]."

The objectives of this paper are to: 1) describe the process we followed to develop the story-based intervention targeted to parents of children with croup; 2) report the feedback received from a sample of parents; and 3) discuss the questions and issues that arose during development. This information will be valuable for further work in the area of storytelling, as well as more broadly in terms of identifying and developing communication strategies for healthcare consumers [22].

## Methods

**Figure 1:**
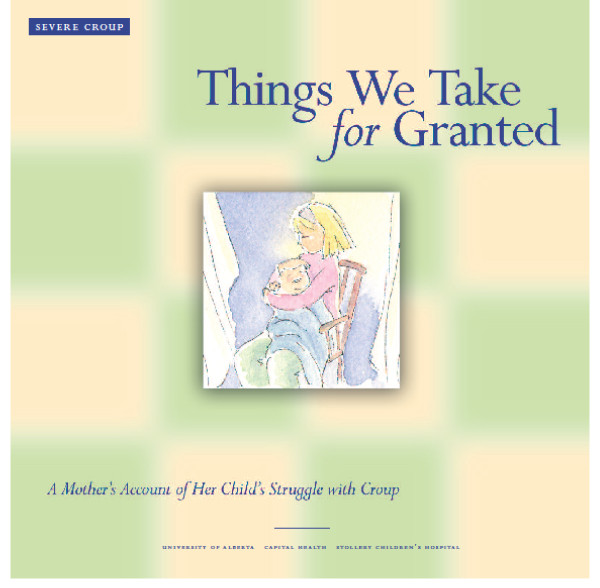
**Cover of one of the story booklets**.

**Figure 2:**
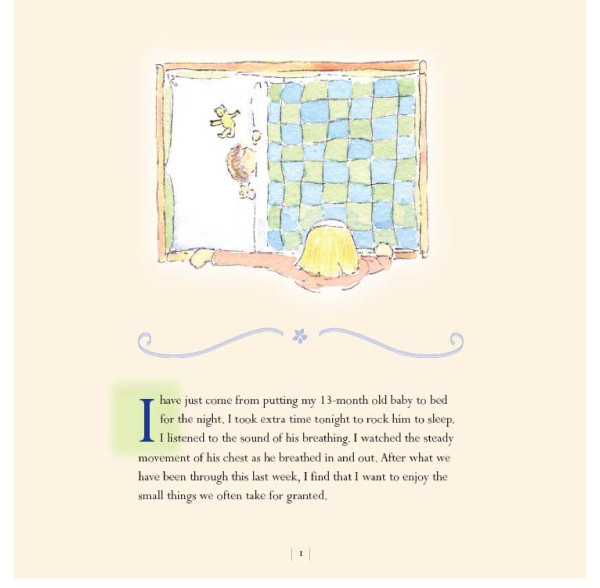
**Excerpt from story booklet**.

**Figure 3:**
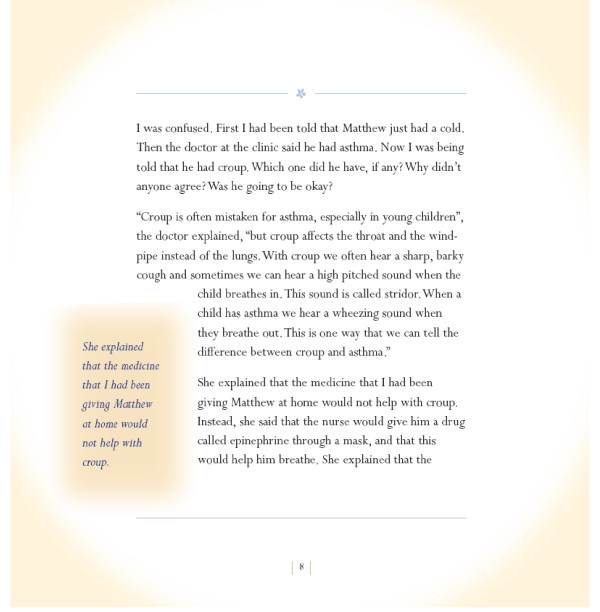
**Excerpt from story booklet**.

**Figure 4:**
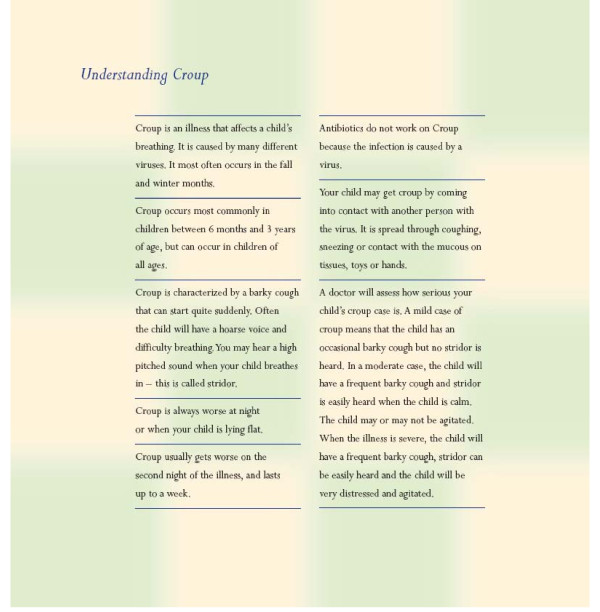
**General information included at the end of the story booklet**.

The storybook intervention was developed through a multi-staged process that began with a creative writer generating the stories (excerpts from one of the stories are presented in Figures 1, 2, 3, and 4). Parent experiences were based on the writer's semi-structured interviews with a sample of families who attended the emergency department (ED) at Alberta Children's Hospital (ACH) with a child presenting with croup between April and September, 2005. The interviews were designed to recount the sequence of events from time of onset of symptoms through to post-ED follow-up, and to elicit the parents' emotional reaction to the experience including their perspectives on the ED management of their child. The creative writer interviewed consenting parents during their ED stay and followed-up by telephone 10 to 14 days after the ED visit to obtain the parents' experiences following discharge. Prior to the interviews, the creative writer was oriented to the ED setting by healthcare professionals on the study team.

Parents/caregivers were eligible if: 1) their child was 3 months to 6 years of age with a clinical diagnosis of croup who was assessed as being eligible for steroids as specified by the Alberta Medical Association Guidelines for croup; 2) they were fluent in English; 3) they were 18 years of age or older; and 4) they had a telephone and would be available for telephone follow-up 10 days after presenting to the ED. The process received ethics approval from the University of Alberta and the University of Calgary and institutional approval from the Stollery Children's Hospital in Edmonton, Alberta and the Alberta Children's Hospital in Calgary, Alberta.

The five stories developed by the creative writer were reviewed by a convenience sample of 10 individuals with a variety of professional and personal backgrounds. One author (LH) revised and edited the stories based on the written feedback and amalgamated the five stories into three. In addition, evidence for the natural history (e.g., signs and symptoms, symptom progression) and medical management (e.g., timing and route of epinephrine and dexamethasone administration) of croup and additional health information (e.g., how and when to contact a healthcare professional, when to seek emergency care) were incorporated either into the story or as part of the booklet. Revising the stories was an iterative process with regular review and feedback by co-authors, including an English professor with expertise in creative non-fiction (TB). The three stories were reviewed for clinical accuracy by three ED physicians and a pediatric nurse.

While there are numerous formats and media to convey stories, *a priori *we chose to develop paper-based story booklets that could be given to parents in the ED. A graphic designer and illustrator created the format and layout and generated illustrations for the booklets in order to complement and enhance the stories. The format and illustrations were critiqued by the study investigators.

The three story booklets were examined through focus groups of parents for presentation, interest, style, and clarity. One author (SS) with relevant experience and expertise in qualitative methodology conducted the focus groups. We identified parents for the focus groups through advertisements posted in numerous locations in Edmonton (Alberta, Canada), including EDs, public health units, medical clinics, and local daycares. We initially aimed to recruit parents of children whose child had experienced croup in the previous year. Due to low numbers, we expanded the eligibility to include any parent with a young child (3-12 years old). The focus groups ran for an hour and participants were reimbursed with Cdn$20. During the focus groups, participants were encouraged to actively and creatively express their views in response to predetermined questions. The questions focused on elements of the storybooks (e.g., length, writing style, accuracy, illustrations) to provide stimulation for discussion and time was allowed for participants to discuss other items or issues that were relevant to them. The focus groups were digitally taped and transcribed verbatim. Interview transcripts were analyzed line by line using content analysis techniques. Ethics approval for the focus groups was obtained from the University of Alberta prior to recruitment.

The booklets were presented at the 2007 Annual Meeting of the Pediatric Emergency Research Canada Network, a national organization of physicians and researchers from pediatric EDs across Canada. The main question for feedback was how to deliver the story booklets to parents, e.g., give all parents all booklets or target by severity of the child's illness.

## Results

The creative writer generated five stories based on her interviews with the parents/caregivers of 10 children presenting to the ED. The five stories were designed to characterize different experiences and cover a range in terms of severity of illness and socioeconomic considerations (e.g., single mother, adolescent mother, Aboriginal background).

The initial written feedback fell into four main categories: 1) overall concept; 2) format and presentation; 3) specific story content; and, 4) medical/health information. Reviewers generally liked the concept of the story booklets and found the project to be interesting and innovative. However, reviewers questioned the specific purpose of the stories (e.g., comfort parents, impart knowledge) and the target audience (i.e., child vs. parent).

Regarding the format, reviewers felt the stories were too long, the language was too advanced for an average reader, and some sentences were complex and awkward. Reviewers liked the illustrations but commented that the font was too small and the titles were "blasé" and did not reflect the main content of the stories. Two reviewers wanted more character development (e.g., more dialogue or more of the characters' thoughts) and more details about the characters and social context. One reviewer commented on the fact that the main character in each of the stories was the mother which may not accurately reflect current parenting roles. Overall, the reviewers found the stories to be engaging largely due to the ability of the writer to capture the parents' emotions.

Several comments were made regarding specific aspects of the stories. For instance, one story described an infant having an x-ray:

"Jimmy had never had an x-ray before, and Diane was not prepared for what she saw. Her heart broke as staff stripped her baby naked and strapped him onto a board which would hold him in place for the x-ray. Though the technicians were very careful with him, Diane was disturbed to see Jimmy crying in the brace. She knew the x-ray was important, but it was the hardest thing she had ever seen as a mom."

Three reviewers found this description to be too harsh and graphic; however, one reviewer thought it was important to prepare a parent for what they might experience. Some incongruencies were noted in the stories (e.g., a 13-month-old being transported in an infant car seat). One reviewer couldn't identify with the main character from one of the stories and another reviewer did not find the introduction to one story captivating; hence, the motivation to read and ability to engage in these stories was compromised.

The final group of comments related to the medical and health information provided in the stories. Reviewers generally wanted as much information as possible about medical procedures and practices and considered the stories to be an excellent potential source of medical advice for parents. Reviewers wanted medical terminology to be explained (e.g., epinephrine mask, dexamethasone) and cautioned against inconsistent use of terminology (e.g., dexamethasone vs. steroid).

Based on this feedback, the stories were substantially revised and reduced from five to three while capturing many of the events and the tone of the original stories. The revised stories were written using simpler language and sentence structure. The revised stories had a Flesch-Kincaid Grade Level Score of 6.2 indicating that a sixth grader (based on US school grade level) could understand them. The three revised stories each reflected a different severity of croup and different healthcare experiences: the mild case was managed at home; the moderate case was seen in the ED and discharged home; and, the severe case was hospitalized for two days. The main characters in the three stories reflected different demographics (e.g., married, single, male, female).

The results of the focus groups were categorized as: 1) general perceptions of the stories; 2) content and emotional by-products of the stories; 3) preferences; and, 4) graphics, layout, and illustrations. The focus group participants were generally very positive about the booklets; however, one participant expressed concerns about the expense of the story booklets and potential wastefulness of healthcare dollars. The participants found the graphics and layout to be visually appealing. They found that they could identify with the stories and that it was easier to get information from the stories compared to a typical information sheet from the ED. The participants suggested that the developer, or sponsor, of the story booklets be more visible to enhance credibility of the content of the books.

Regarding the content of the stories, the participants generally found the stories interesting, engaging, and easy to read. They found that the stories resonated with them and "matched" their personal experience. They found the information to be very helpful and appreciated the suggestions in the stories, specifically how to cope with having a child with croup. The participants found the stories to provide comfort and emotional reassurance. They commented that explaining the rationale for treatments would be useful (e.g., why cold air helps). The participants highlighted some errors (e.g., use of term web browser rather than search engine, inconsistencies in facts presented at the back of the books, typographical errors).

One issue relating to story content generated much discussion and consideration: in one of the stories, the parents did not take the child to the ED but managed the child's symptoms at home based on information they found through the internet. The participants suggested providing in the story booklets a list of recommended websites or information on how to evaluate websites and whether they are a trustworthy source of information.

The focus group participants highlighted several preferences. The parents generally preferred one story as they found they could relate most to this story; in their words, they saw themselves in the story. They appreciated the fact that the story was written in the first-person mode as this format held their attention better. The participants appreciated having a father as the main character in one of the stories and the fact that he accessed the internet for information. Finally, the participants liked the "catchy" titles of the stories.

We found little guidance in the literature in terms of presentation styles (e.g., size and placement of illustrations, font size and colour, other layout considerations, shape and size of booklets); hence, the information gained from the parent focus groups was very informative. The focus group participants found the presentation of the booklets soft and eye appealing. They enjoyed the variety in the illustrations (e.g., some full page, some half page). The participants all preferred the booklet that was the same shape and size as many children's books and considered this as a positive feature. The participants felt that the illustrations could appeal to both adults and children, thereby serving as a method for parents to explain to their child what may happen in the ED. The participants enjoyed the illustrations and the colours used throughout the books; however, in one case the use of different coloured font for portions of the text created confusion for the reader regarding what was or was not important to read.

We revised the story booklets based on the focus group feedback and presented the final products at a national conference of pediatric emergency clinicians and researchers. The primary question for this group was how to package and disseminate the story booklets. The general consensus was to provide the three booklets to parents in a single package. We developed a folder for this purpose which held the three booklets.

## Discussion

We followed a thorough and extensive process to develop the story booklets to provide information and comfort parents attending the ED with a child with croup. The intent was to develop an intervention for use in a randomized controlled trial to evaluate the effectiveness of storytelling as a communication tool. We found that the development of such an intervention involved numerous decisions which were best informed through involvement of the end-user group. In general the feedback was very positive, although one focus group participant questioned the costs involved in producing the story booklets and whether the resources would be better spent elsewhere. A recurring theme of the feedback was the ability of the reader to relate to the stories and identify with the characters, which has been identified as a key factor for stories to be effective [20,23]. In fact one reviewer said the stories brought tears to her eyes as she recalled her own similar experiences. The specific feedback in terms of story content, errors and inconsistencies, and presentation style was critical for accuracy and in order to target the story booklets to the end-users' needs and preferences. Our key findings are summarized in Table 1.

**Table 1:** Summary Points

**What is already known on this topic:**
*There has been investigation into the use of narrative and stories as a tool to communicate health information to consumers.
*There are few reports describing the development of narrative or story-based interventions including focus groups involving the end-user group.
**What this study adds:**
*We followed a systematic process to develop a story-based intervention for parents attending the emergency department for the care of children with an acute, self-limiting condition.
*The parent focus groups provided rich feedback and allowed us to shape our products to ensure accuracy, credibility, and relevance to the end-user.
*Our experience highlights many considerations for future development work in this area, and more broadly for patient education materials, including clear identification of the purpose and goals of the end-product at the outset and involvement of the end-user group throughout to identify needs and preferences.

There were several challenges we encountered during the development of the story booklets. A key challenge, highlighted by one of the initial reviewers, was regarding the purpose of the story booklets. We hypothesized that the story booklets could serve a number of purposes, such as communicating information, contextualizing the illness experience and medical encounter, providing a decision aid, and building relationships between healthcare providers and parents/patients or among parents/patients undergoing the same experiences; however, our primary purpose was to provide information and comfort to parents in an effort to reduce parental anxiety. We considered this a critical initial step and would encourage others engaged in similar work to carefully consider the purpose of their intervention (and related primary outcomes) and what they plan to achieve through both product development and utilization. This is critical not only for the development of the interventions, but also to evaluate their effectiveness. The purpose of the intervention should be directly related to the outcomes to be assessed in its evaluation: e.g., communicating information (recall of information, satisfaction with information, compliance with information or instructions); contextualizing (comfort, anxiety); decision aid (decision regret, comfort/ease of decision-making, or subsequent resource use); and, building relationships (feelings of being supported, satisfaction).

A second challenge was staying true to the story vs. being evidence-based. For instance, in one case the child was given an x-ray, despite the fact that this is not standard practice for croup and does not conform to accepted clinical practice guidelines. Our dilemma was whether to recount the events as described by this parent or reflect accepted clinical practice in the form of composite narratives. In the end, we did not include the x-ray account and aimed to make the stories reflect typical cases of mild, moderate, and severe disease and how they would be managed on average. Another example was a situation where a child had been misdiagnosed prior to the ED visit. We had to consider whether it was appropriate to point out that physicians may make errors in diagnosis. We decided to include the incident in the story to highlight a relatively common error of misdiagnosing croup for asthma. It also provided an opportunity in the story to educate the parents around the differences of croup and asthma, and particularly the different treatments appropriate for each.

Another dilemma regarding being evidence-based was whether or not to describe interventions for which there was no evidence. For instance, there is widespread practice and recommendations around the use of mist or humidity for croup despite no research evidence supporting its effectiveness. A further issue was around the naming of drugs in the stories and the potential perception of product placement. For example, many parents would be more familiar with "Tylenol" than "acetaminophen." We chose to use the trade names that are more familiar to the lay person but included a range of product names to not appear preferential to a single brand.

A related issue was how much additional information or evidence to incorporate into the stories. Many of the reviewers wanted more detailed medical information; however, this was not typically captured in the parents' recounting of events. In the end, we included a fair amount of information about croup and its management (e.g., signs and symptoms, what is a steroid, how the drugs are administered), but tried to incorporate this detail as seamlessly as possible into the story while preserving its narrative flow and tone. We also felt bound ethically to provide appropriate information on when to seek additional or emergency care. Further, based on feedback we added a forward to each of the story booklets by the ED director of the local children's hospital as an endorsement of the story booklets by the healthcare system.

An issue that generated much discussion and controversy was the parents' reliance in one of the stories on information found on the internet. This raised concerns that we may inappropriately condone information that is found on the internet, as well as ethical concerns that we may be encouraging parents to manage their child's illness at home when the child may require medical care. Nevertheless, we recognized that lay people are regularly using the internet as a source of medical information and felt that it was an important issue to profile in our stories. We addressed these concerns in several ways. First, we reviewed many of the websites that were identified when searching Google for "croup." Because the evidence is so strong for the management of croup, the information across websites was very consistent and accurate. Second, we suggested a website, that we know to provide reliable information, within the stories that parents could go to for additional information. Third, we included information at the end of each of the stories about when to seek medical care and specifically when to seek emergency care.

A major challenge was developing stories that would be widely generalizable and appealing [24]. Numerous considerations arose such as how many stories, story length, reading level, narrative mode (e.g., first person, third person), representation of different demographics (e.g., sex, race, age, socioeconomic status), and representation of different illness experiences (e.g., severity of illness, hospitalization, management at home). We had to strike a delicate balance between being as inclusive, generalizable, and detailed as possible [23], while being as succinct as possible to increase the likelihood that parents would read the complete stories [20,25]. These considerations need to be informed by the end-user group, specifically those who are most likely to benefit from the intervention.

A major consideration and investment of resources related to the presentation and packaging of the stories. There are many media through which stories can be delivered (e.g., computer, video, games, cartoons, etc.). *A priori *we chose for this project to use paper-based storybook formats. However, there remained many considerations within this format such as the type of illustrations, use of graphics and colour, and shape and size of the booklets [26]. Again, the feedback we received through the parent focus groups was helpful in making decisions. The format of delivering stories is particularly dependent on the preferences of the target end-users.

While the feedback we gathered was rich and informative, our process was limited by the number of individuals that we were able to recruit for our focus groups. We advertised widely across numerous venues and had very few willing participants. We found it particularly challenging to recruit as our target end-user was parents with young children who have many competing priorities and time constraints.

## Conclusions

We followed a systematic process to develop story booklets for parents attending the ED with a child with croup. The parent focus groups provided rich feedback and allowed us to shape our products to ensure accuracy, credibility, and relevance to the end-user. Our experience highlights many considerations for future development work in this area, including clear identification of the purpose and goals of the end-product at the outset and involvement of the end-user group throughout to identify needs and preferences. Moreover, our results are informative more broadly for the development of patient education materials and tools to communicate with patients. Whether the story booklets are effective in practice needs to be assessed using rigorous, research methods. A randomized controlled trial is currently underway with a concomitant qualitative study to assess the attributes of the stories that facilitate successful knowledge transfer to parents.

## Competing interests

The authors declare that they have no competing interests.

## Authors' contributions

LH contributed to the design of the study, revised and edited the stories, interpreted results, and drafted the manuscript. SS contributed to the design and coordination of the study, critically reviewed the stories, conducted the focus groups and analyzed focus group data, and drafted relevant sections of the manuscript. RP coordinated the project, critically reviewed the stories, and provided feedback on the manuscript. DJ contributed to the design and implementation of the study, provided critical feedback on the stories, and provided feedback on the manuscript. TB provided critical feedback on the stories and reviewed the manuscript. TPK conceived of the initial idea, contributed to the design and implementation of the study, and provided feedback on the manuscript. All authors read and approved the final manuscript.

## Funding

This study was funded in part by a Team Grant in Pediatric Emergency Medicine from the Canadian Institutes of Health Research. The researchers had independence from the funder in all aspects of the research.

## Pre-publication history

The pre-publication history for this paper can be accessed here:

http://www.biomedcentral.com/1471-2431/10/64/prepub
